# Inhibitory Mechanism of *FAT4* Gene Expression in Response to Actin Dynamics during Src-Induced Carcinogenesis

**DOI:** 10.1371/journal.pone.0118336

**Published:** 2015-02-13

**Authors:** Takao Ito, Hiroaki Taniguchi, Kousuke Fukagai, Shota Okamuro, Akira Kobayashi

**Affiliations:** Laboratory for Genetic Code, Graduate School of Life and Medical Sciences, Doshisha University, Kyotanabe, Kyoto, Japan; Wayne State University, UNITED STATES

## Abstract

Oncogenic transformation is characterized by morphological changes resulting from alterations in actin dynamics and adhesive activities. Emerging evidence suggests that the protocadherin FAT4 acts as a tumor suppressor in humans, and reduced *FAT4* gene expression has been reported in breast and lung cancers and melanoma. However, the mechanism controlling *FAT4* gene expression is poorly understood. In this study, we show that transient activation of the Src oncoprotein represses *FAT4* mRNA expression through actin depolymerization in the immortalized normal human mammary epithelial cell line MCF-10A. Src activation causes actin depolymerization via the MEK/Erk/Cofilin cascade. The MEK inhibitor U0126 blocks the inhibitory effect of Src on *FAT4* mRNA expression and Src-induced actin depolymerization. To determine whether actin dynamics act on the regulation of *FAT4* mRNA expression, we treated MCF-10A cells with the ROCK inhibitor Y-27632. Y-27632 treatment decreased *FAT4* mRNA expression. This suppressive effect was blocked by siRNA-mediated knockdown of Cofilin1. Furthermore, simultaneous administration of Latrunculin A (an actin depolymerizing agent), Y-27632, and Cofilin1 siRNA to the cells resulted in a marked reduction of *FAT4* mRNA expression. Intriguingly, we also found that *FAT4* mRNA expression was reduced under both low cell density and low stiffness conditions, which suggests that mechanotransduction affects *FAT4* mRNA expression. Additionally, we show that siRNA-mediated FAT4 knockdown induced the activity of the Hippo effector YAP/TAZ in MCF-10A cells. Taken together, our results reveal a novel inhibitory mechanism of *FAT4* gene expression through actin depolymerization during Src-induced carcinogenesis in human breast cells.

## Introduction

Oncogenic cell transformation results from the summation of changes in cell growth, cell viability, cell motility and cell morphology. The v-Src oncogene, a product of the Rous sarcoma virus, is the constitutively active form of c-Src. Src has the ability to regulate various signal transduction pathways, including the Ras/MEK/Erk, PI3K/Akt, STAT3, and Rho/ROCK pathways [1–4]. More specifically, Src has been reported to induce alterations in cell morphology through actin dynamics and to depolymerize the actin cytoskeleton via the MEK/Erk/Cofilin cascade [5]. Additionally, members of the cadherin superfamily have been implicated in Src-induced tumor transformation. Src downregulates E-cadherin expression and triggers morphological changes in multiple cancers [6–8]. These findings suggest the importance of both actin dynamics and the loss of cadherin-mediated cell-cell adhesion in Src-induced tumorigenesis.

FAT4, a protocadherin, is the human ortholog of *Drosophila* Fat [9,10]. Recent studies indicate that *Drosophila* Fat suppresses tumorigenesis through activation of the Hippo pathway. In support of this finding, PrognoScan, a new microarray database [11], and other recent studies [12–14] have demonstrated that human *FAT4* gene expression is repressed in breast and lung cancers and in melanoma, which suggests that reduced *FAT4* gene expression can trigger carcinogenesis. However, the molecular mechanisms underlying the down-regulation of *FAT4* gene expression in human cancers remain unknown.

The Hippo pathway is involved in tumor suppressor signaling and regulates organ size, cell proliferation, apoptosis and stemness [9,10,15,16]. In mammals, the core components of the Hippo pathway are primarily composed of Mst1/2 and Lats1/2, which inactivate the Hippo effector Yap/Taz via phosphorylation [17–22]. Yap/Taz acts as a transcriptional co-activator by binding the transcription factors Tead1–4 to induce the expression of genes such as the connective tissue growth factor CTGF [18,23,24]. Fat4 has been shown to repress Yap activity and thereby negatively regulates neural progenitor cell proliferation during mammalian neurogenesis [25,26]. Thus, the FAT4-Hippo axis is believed to be functionally conserved in humans; however, the functional significance and the molecular mechanisms that confer this axis are poorly understood.

Here, we show that transient Src activation represses *FAT4* mRNA expression and leads to tumor transformation in MCF-10A cells, which are immortalized normal human mammary epithelial cells. We further demonstrate that the inhibitory effect of Src on *FAT4* mRNA expression depends on actin depolymerization that is induced by the MEK/Erk/Cofilin cascade. Finally, we demonstrate that FAT4 represses YAP/TAZ activity in MCF-10A cells.

## Results

### *FAT4* gene expression is repressed during human breast cell transformation by the Src oncogene

**Fig 1:**
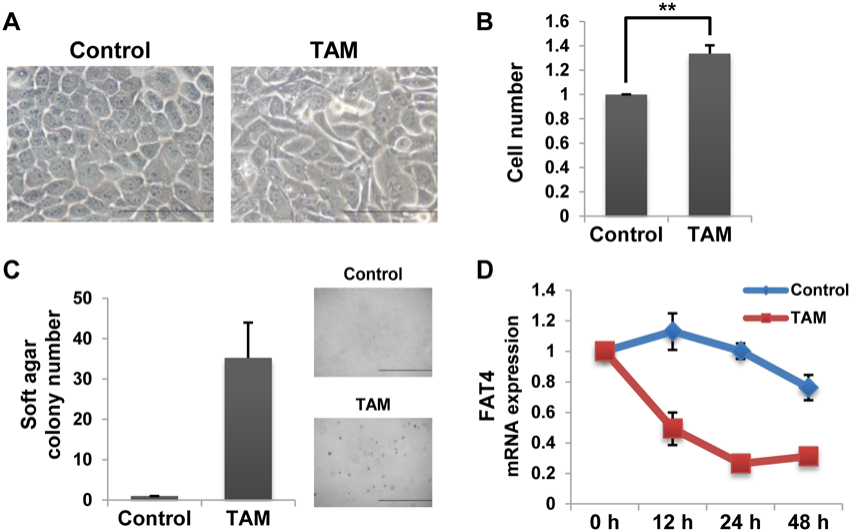
Transient activation of Src depresses *FAT4* gene expression and induces tumor transformation in MCF-10A cells. **A**. Images show morphological changes of MCF-10A v-Src:ER cells treated with 1 μM TAM for 36 h. Black bars, 100 μm. **B**. WST-1 Assay in TAM-treated cells (48 h) (mean ± SD, n = 3, ** indicates *P* < 0.01). **C**. Soft Agar Colony Formation Assay in TAM-treated cells (72 h) (mean ± SD, n = 4). Images show cell colonies. Black bars, 1 mm. **D**. RT-qPCR analysis of *FAT4* mRNA expression levels in TAM-treated cells for the indicated times (mean ± SD, n = 3).

To analyze the mechanisms that repress *FAT4* gene expression in human breast cancer, we generated a model of carcinogenesis using MCF-10A cells, an immortalized normal human mammary epithelial cell line [27–31]. In brief, we used retroviruses to create MCF-10A cells that stably express the Src kinase oncoprotein (v-Src) fused to the ligand binding domain of the estrogen receptor (ER). In MCF-10A v-Src:ER cells, the addition of 4-Hydroxytamoxifen (TAM) induces Src activation and their subsequent cell transformation. TAM treatment resulted in morphological transformation (Fig. 1A), enhanced cell proliferation (Fig. 1B), and promoted anchorage-independent growth (Fig. 1C). In this model, we found that *FAT4* mRNA expression was repressed by Src activation in MCF-10A cells (Fig. 1D). On the other hand, *TWIST1* mRNA expression was not altered under similar experimental conditions (S1 Fig.). This points to the specificity of the inhibitory effect of Src on *FAT4* mRNA expression. Thus, these results indicate that Src induces tumorigenesis and suppresses *FAT4* mRNA expression in MCF-10A cells.

### Src activation induces both *FAT4* gene repression and Cofilin-mediated actin depolymerization through the MEK/Erk pathway

**Fig 2:**
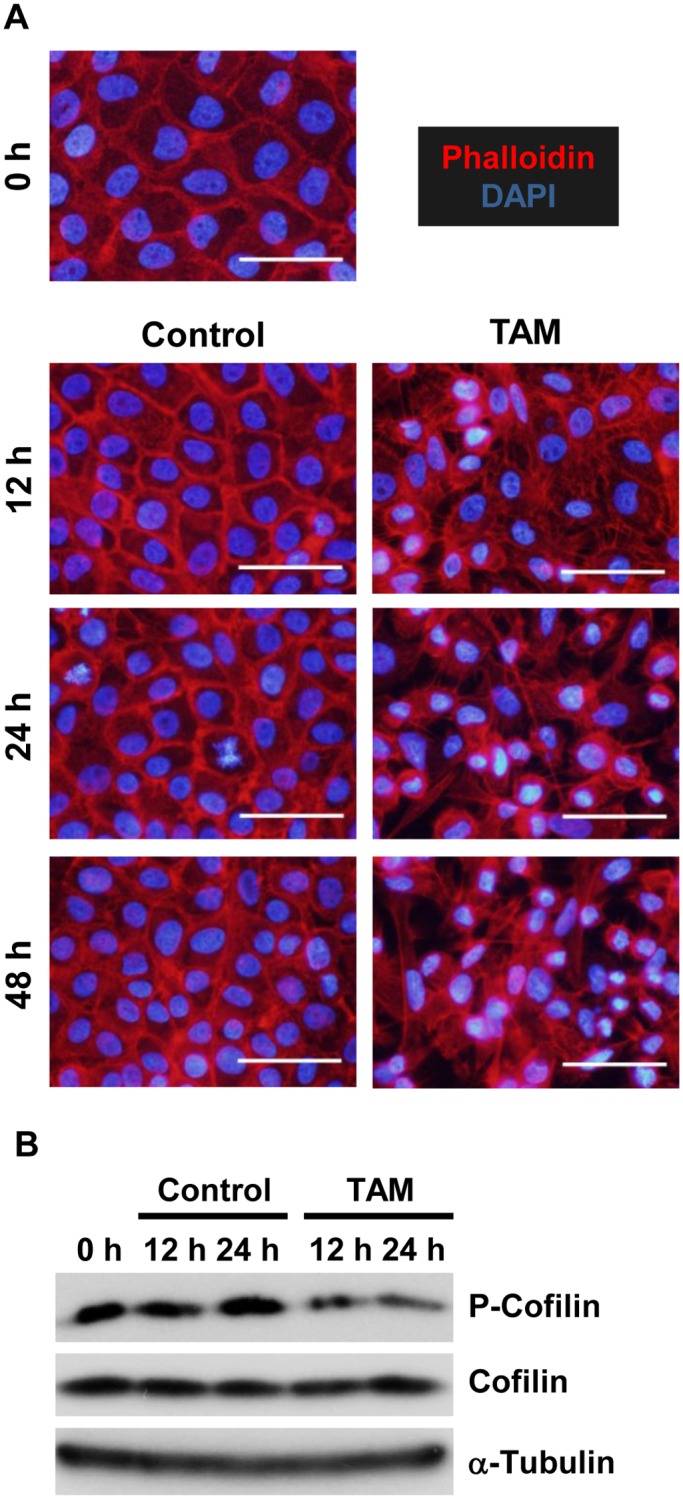
Src depolymerizes actin filaments via dephosphorylation of Cofilin. **A**. Staining for actin filaments (F-actin) (Phalloidin) and nuclei (DAPI) in TAM-treated MCF-10A v-Src:ER cells. The cells were treated with 1 μM TAM for the indicated time periods. White bars, 50 μm. **B**. Western blotting for phosphorylated and total Cofilin (P-Cofilin and Cofilin, respectively) in TAM-treated cells for the indicated time periods. Western blotting for α-Tubulin was used as a loading control.

**Fig 3:**
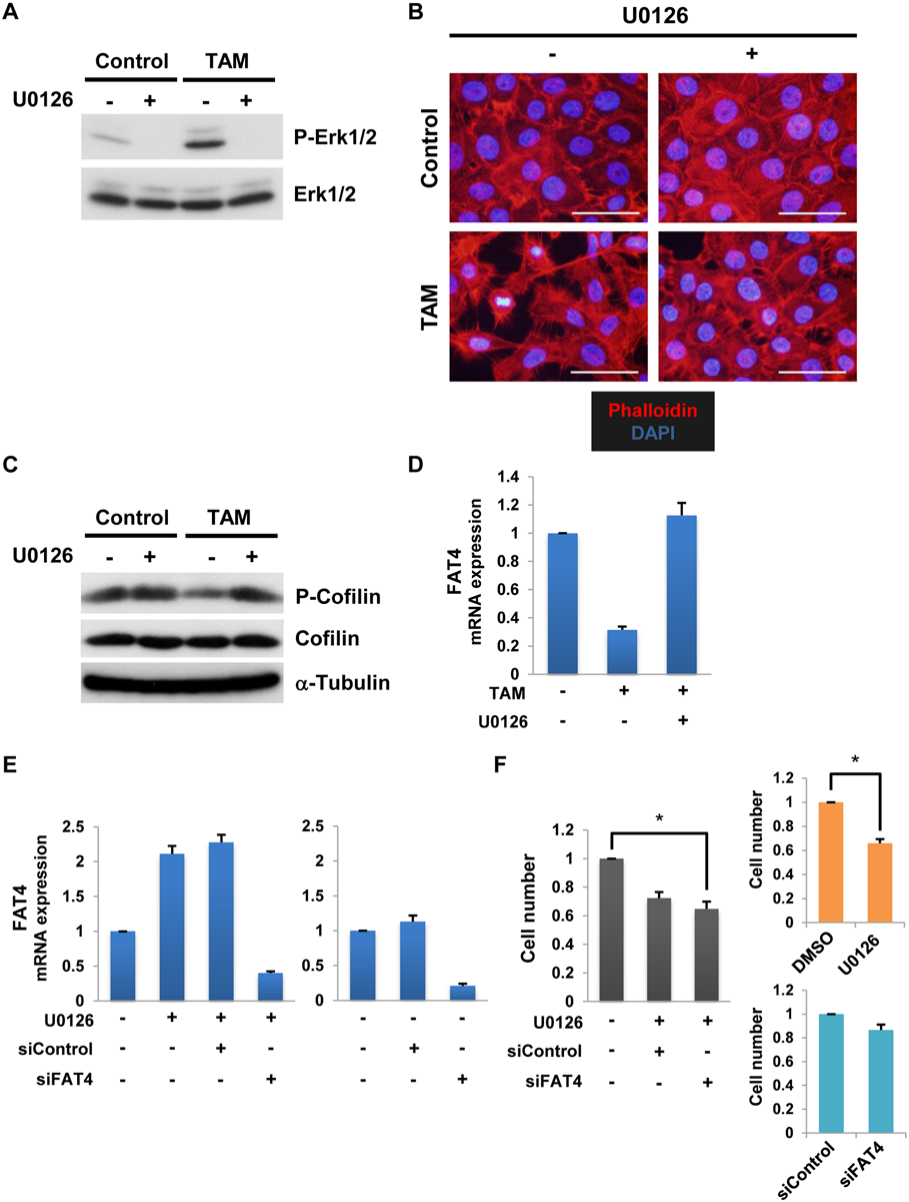
The MEK/Erk pathway is indispensable for both *FAT4* gene repression and Cofilin-mediated actin depolymerization by Src. **A**. Western blotting for phosphorylated and total Erk1/2 (P-Erk1/2 and Erk1/2, respectively) in MCF-10A v-Src:ER cells. Serum-starved cells (16 h) were cultured in the presence or absence of the MEK inhibitor U0126 for 1 h (30 μM) and then treated with 1 μM TAM for 1 h. **B**. Staining for F-actin (Phalloidin) and nuclei (DAPI) in TAM-treated cells (24 h) after pretreatment with 30 μM U0126 for 1 h. White bars, 50 μm. **C**. Western blotting for the indicated proteins in TAM-treated cells after pretreatment with U0126. **D**. RT-qPCR analyses of *FAT4* mRNA expression levels in TAM-treated cells after pretreatment with U0126 (mean ± SD, n = 3). **E**. RT-qPCR analyses of *FAT4* mRNA expression levels in MDA-MB-231 cells treated with 20 μM U0126 for 24 h after pretreatment with control or FAT4 siRNA (siControl and siFAT4, respectively, 20 nM) (mean ± SD, n = 3). **F**. WST-1 Assay in U0126-treated MDA-MB-231 cells (48 h) after pretreatment with siFAT4 for 24 h (mean ± SD, n = 5, * indicates *P* < 0.05).

Src has been shown to elicit Cofilin-mediated disruption of the actin cytoskeleton through the MEK/Erk pathway [5]. The function of Cofilin is activated via its dephosphorylation [32]. Thus, we examined whether Src can disrupt the actin cytoskeleton through dephosphorylation of Cofilin in MCF-10A v-Src:ER cells. Indeed, phalloidin staining and Western blot analysis demonstrated that Src can depolymerize the actin cytoskeleton and dephosphorylate Cofilin (Fig. 2). We further examined the involvement of the MEK/Erk pathway in Src-mediated modulation of the actin cytoskeleton. Treatment of MCF-10A v-Src:ER cells with the MEK inhibitor U0126 completely inhibited Src-mediated phosphorylation of Erk1/2, a major target of MEK1/2 (Fig. 3A). Under this condition, U0126 significantly inhibited both Src-induced actin dynamics (Fig. 3B) and the dephosphorylation of Cofilin (Fig. 3C). Therefore, these results indicate that Src induces Cofilin-mediated actin depolymerization through the MEK/Erk pathway in MCF-10A cells.

We also investigated whether the MEK/Erk pathway is involved in Src-reduced *FAT4* mRNA expression by RT-qPCR analysis; the results suggest that Src-mediated inhibition of *FAT4* mRNA expression is repressed in MCF-10A cells by U0126 or PD0325901, a MEK inhibitor (Fig. 3D and S2 Fig.). In addition, to emphasize the importance of the MEK/Erk pathway in *FAT4* mRNA repression we evaluated the effect of U0126 on *FAT4* mRNA expression in MDA-MB-231 cells, a malignant breast cancer cell line. Treatment of MDA-MB-231 cells with U0126 also up-regulated *FAT4* mRNA expression (Fig. 3E). Thus, these results indicate that Src represses *FAT4* mRNA expression through the MEK/Erk pathway. Intriguingly, siRNA-mediated knockdown of FAT4 did not block U0126-induced growth inhibition of MDA-MB-231 cells (Fig. 3F), perhaps because the MEK/Erk pathway regulates various targets that include *FAT4* gene. Collectively, these results suggest that Src activation induces not only Cofilin-mediated actin depolymerization but also *FAT4* mRNA repression through the MEK/Erk pathway.

### Src represses *FAT4* gene expression through Cofilin1-mediated actin depolymerization

**Fig 4:**
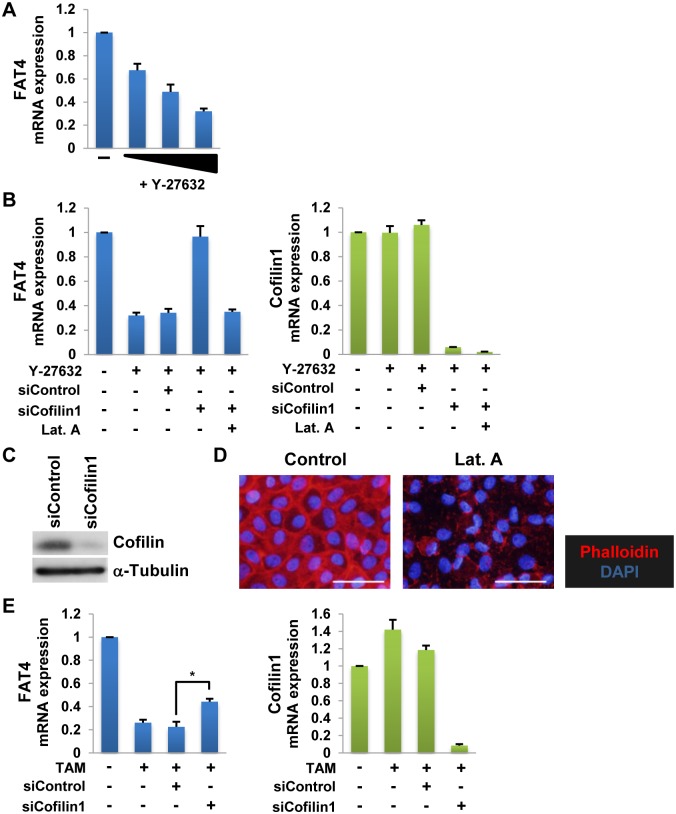
Src represses *FAT4* gene expression through Cofilin1-mediated actin depolymerization. **A**. RT-qPCR analyses of *FAT4* mRNA expression levels in MCF-10A cells treated with the ROCK inhibitor Y-27632 (10, 30 and 50 μM) for 24 h (mean ± SD, n = 3). **B**. RT-qPCR analyses of *FAT4* and *Cofilin1* mRNA expression levels in MCF-10A cells treated with 50 μM Y-27632 and 0.5 μM Latrunculin A (Lat. A) for 24 h after pretreatment with control or Cofilin1 siRNA (siControl and siCofilin1, respectively, 20 nM) (mean ± SD, n = 3). **C**. Western blotting for the indicated proteins in cells transfected with siCofilin1 for 24 h. **D**. Staining for F-actin (Phalloidin) and nuclei (DAPI) in Lat. A-treated cells (24 h). White bars, 50 μm. **E**. RT-qPCR analyses of *FAT4* and *Cofilin1* mRNA expression levels in TAM-treated MCF-10A v-Src:ER cells (24 h) after pretreatment with siCofilin1 (70 nM) (mean ± SD, n = 3, * indicates *P* < 0.05).

Next, we examined the causal relationship between *FAT4* mRNA repression and Cofilin-mediated actin depolymerization, both of which are downstream of the Src/MEK/Erk pathway. The ROCK pathway is a major pathway that regulates Cofilin-mediated actin depolymerization [33,34]. Thus, to determine whether Src downregulates *FAT4* mRNA expression by altering actin structures, we examined the effect of the ROCK inhibitor Y-27632 on *FAT4* mRNA expression. Treatment of MCF-10A cells with Y-27632 reduced *FAT4* mRNA expression in a dose-dependent manner (Fig. 4A). The inhibitory effect of Y-27632 on *FAT4* mRNA expression was abrogated by siRNA-mediated knockdown of Cofilin1 (Fig. 4B). Cofilin1 is a non-muscle type of Cofilin [32]. Effective knockdown of Cofilin1 was supported by RT-qPCR (Fig. 4B) and Western blot analysis (Fig. 4C). Moreover, simultaneous administration of the actin depolymerizing agent Latrunculin A, Y-27632, and siCofilin1 to the cells resulted in a striking decrease of *FAT4* mRNA expression equal to that of the Y-27632 treatment alone (Fig. 4B and S3 Fig.). Phalloidin staining showed that Latrunculin A sufficiently depolymerizes the actin cytoskeleton (Fig. 4D). Therefore, on one hand, these results suggest that ROCK1/2 regulates *FAT4* mRNA expression via Cofilin1-mediated actin depolymerization. On the other hand, they suggest that inhibition of Cofilin1 significantly attenuates *FAT4* mRNA repression by Src (Fig. 4E). Therefore, we propose that Src suppresses *FAT4* mRNA expression through actin depolymerization by Cofilin1.

### Mechanotransduction affects *FAT4* gene expression

**Fig 5:**
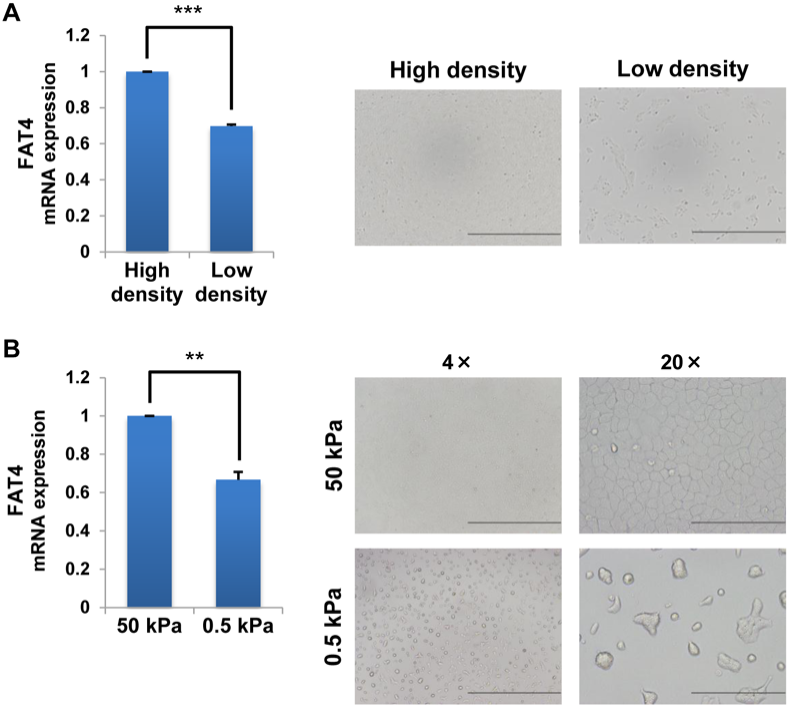
Mechanotransduction affects *FAT4* gene expression. **A**. Cell Density Assay. RT-qPCR analyses of *FAT4* and *CTGF* mRNA expression in MCF-10A cells grown under high or low cell density conditions for 24 h (4.2×10^5^ cells and 7.0×10^4^ cells per well on a 6-well plate, respectively) (mean ± SD, n = 3, *** indicates *P* < 0.001). Images show cells grown under high or low cell density conditions for 24 h. Black bars, 1 mm. **B**. Softwell Assay. RT-qPCR analyses of *FAT4* and *CTGF* mRNA expression in MCF-10A cells grown on stiff (50 kPa) or soft (0.5 kPa) 10 μg/ml Collagen I-coated Softwell plates for 48 h (mean ± SD, n = 3, ** indicates *P* < 0.01). Images show cells grown on the indicated stiffness for 24 h. Black bars, 1 mm or 200 μm.

Recent studies have reported that mechanical stress on cells affects cellular behavior via the modulation of actin dynamics [35–37]. Based on our findings that *FAT4* mRNA expression is regulated by actin depolmerization through Cofilin1 (Fig. 4), we examined whether mechanical stress also affects *FAT4* mRNA expression. To test this hypothesis, we performed a Cell Density Assay and a Softwell Assay. In the Cell Density Assay, MCF-10A cells were seeded at high and low cell densities to evaluate the effect of cell-cell contact on *FAT4* mRNA expression. The Softwell Assay used collagen type I-coated Softwell plates of varying degrees of stiffness (50 kPa and 0.5 kPa) to assess the effect of anchorage stiffness on *FAT4* mRNA expression. *FAT4* mRNA expression was reduced under both low cell density (Fig. 5A) and low anchorage stiffness conditions (Fig. 5B). These findings suggest that the mechanical stress that stems from the extracellular environment is intimately linked with *FAT4* mRNA expression. Moreover, we found no significant difference in phoshorylated-Cofilin expression between high and low density cells (S4 Fig.). Thus, it may be assumed that the observed alteration in *FAT4* mRNA expression under low density conditions is not mediated through the MEK/ERK/Cofilin cascade.

### FAT4 suppresses the activity of the Hippo effector YAP/TAZ

**Fig 6:**
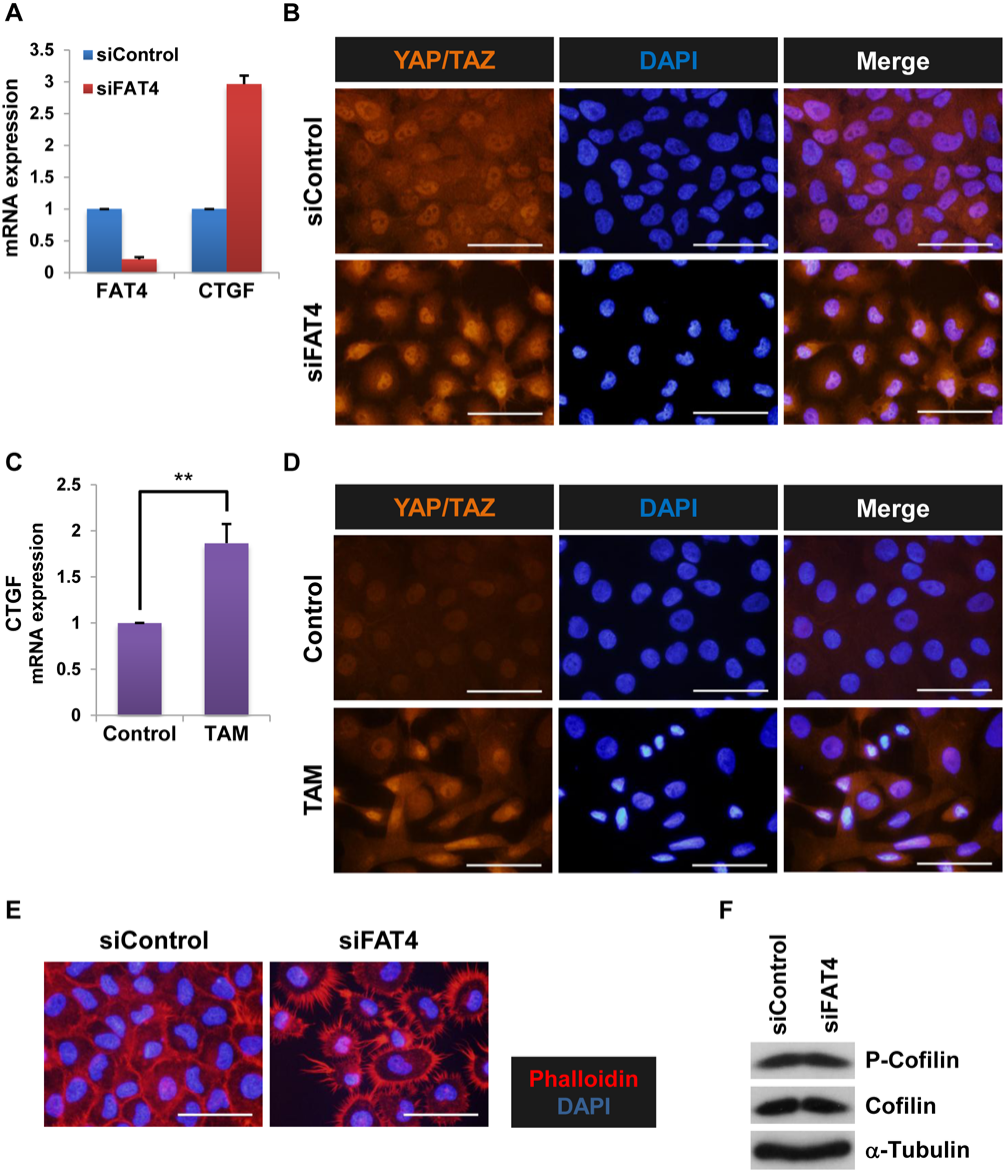
FAT4 negatively regulates the activity of the Hippo effector YAP/TAZ. **A**. RT-qPCR analyses of *FAT4* and *CTGF* mRNA levels in MCF-10A cells transfected with control or FAT4 siRNA (siControl and siFAT4, respectively, 30 nM) for 48 h (mean ± SD, n = 3). **B**. Immunofluorescence images of YAP/TAZ in MCF-10A cells transfected with siFAT4 for 48 h. The nuclei were stained with DAPI. White bars, 50 μm. **C**. RT-qPCR analysis of *CTGF* mRNA expression levels in 24 h TAM-treated MCF-10A v-Src:ER cells (mean ± SD, n = 3, ** indicates *P* < 0.01). **D**. Immunofluorescence images of YAP/TAZ in TAM-treated cells (24 h). The nuclei were stained with DAPI. White bars, 50 μm. **E**. Staining for F-actin (Phalloidin) and nuclei (DAPI) in cells transfected with siFAT4 for 48 h. White bars, 50 μm. **F**. Western blotting for the indicated proteins in 48 h siFAT4-treated cells.

Accumulating evidence suggests that the FAT4-Hippo axis is also conserved in humans [12–14,25,26]. We next examined the effects of FAT4 knockdown on the function of the Hippo effector YAP/TAZ. YAP/TAZ activity is suppressed by cytoplasmic retention and proteasomal degradation due to LATS1/2-mediated phosphorylation of YAP/TAZ [38–42]. FAT4 siRNA increased mRNA expression levels of *CTGF*, a YAP/TAZ target gene (Fig. 6A) [18–21,23,24], by promoting the nuclear accumulation of YAP/TAZ (Fig. 6B). Furthermore, we found that the oncoprotein Src promoted YAP/TAZ activity in MCF-10A cells (Figs. 6C and 6D). These results are consistent with those of a recent study that demonstrated *Drosophila* Src64B activates Yorkie, the mammalian Yap/Taz homologue, by repressing the core components of the Hippo pathway (Hippo/Warts) [43]. Thus, our results suggest that FAT4 represses *CTGF* mRNA expression through the inactivation of YAP/TAZ.

We next examined the molecular basis of YAP/TAZ activation by FAT4 knockdown. Because YAP/TAZ activity is regulated by mechanotransduction via actin dynamics [35,36,38], we investigated the effects of FAT4 siRNA on actin dynamics. Phalloidin staining revealed that FAT4 knockdown induced abnormal spiny actin structures that radiated in all directions from the cells (Fig. 6E and S5 Fig.). However, this structural modulation of actin was independent of Cofilin because we observed no increase in phosphorylated Cofilin (Fig. 6F). Taken together, our results may suggest that FAT4 represses YAP/TAZ activity by regulating actin structures.

### FAT4 knockdown does not induce cell transformation

Finally, we investigated whether FAT4 knockdown alone can transform MCF-10A cells because FAT4 knockdown activates YAP/TAZ, which elicits cell transformation (Figs. 6A and 6B) [23,24]. However, FAT4 knockdown alone did not cause increased cell proliferation nor anchorage-independent growth in MCF-10A cells (S6 Fig.). Therefore, combinatorial effects of reduced *FAT4* gene expression and other factors could be necessary for tumor transformation in MCF-10A cells.

## Discussion

**Fig 7:**
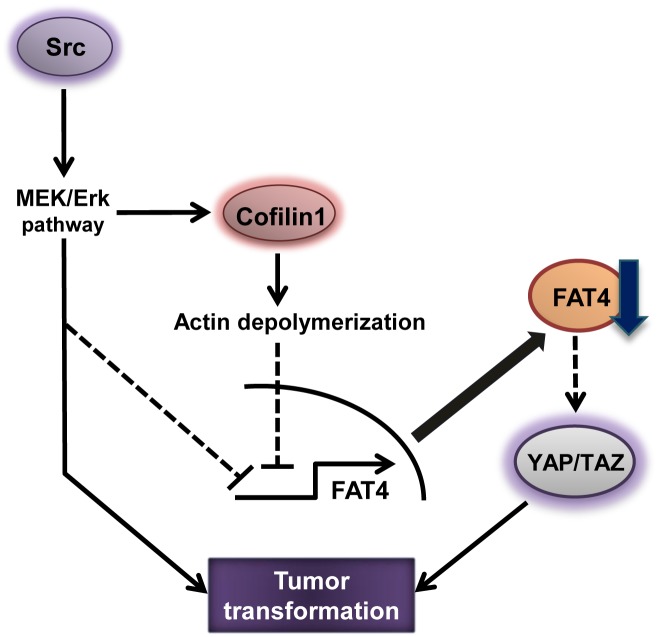
A model of *FAT4* gene repression in Src-induced tumorigenesis. Src activation represses *FAT4* gene expression through the MEK/Erk/Cofilin1 cascade, which induces actin depolymerization in tumor transformation. *FAT4* gene repression further elicits YAP/TAZ activation.

Reduced *FAT4* gene expression in human breast cancers has been reported in several studies [11,12]. Nevertheless, the molecular mechanism that governs this down-regulation in gene expression remains unknown. Here, we show that *FAT4* mRNA expression is repressed in response to actin dynamics during Src-induced tumor transformation of MCF-10A cells (Fig. 7). Src-mediated actin depolymerization is regulated by the MEK/Erk/Cofilin1 cascade and results in the repression of *FAT4* mRNA expression. Finally, we demonstrate that reduced expression of FAT4 induces the activity of the Hippo effector YAP/TAZ in MCF-10A cells. Altogether, these results propose a novel mechanism by which actin dynamics regulate cancer-related traits through the loss of FAT4-mediated cell-cell adhesion.

In this study, we demonstrate that the Src/MEK/Erk/Cofilin1 cascade, which mediates actin depolymerization, represses *FAT4* gene expression in MCF-10A cells (Fig. 7). The mechanism by which the MEK/Erk pathway represses *FAT4* gene expression is conserved in even the malignant breast cancer cell line MDA-MB-231 cells as well as the normal breast cell line MCF-10A cells (Fig. 3D and 3E). Src activates Cofilin via dephosphorylation by components of the MEK/Erk pathway (Fig. 3C) and thereby induces actin depolymerization (Fig. 3B). Notably, the MEK/Erk pathway plays an important role in activating Cofilin in a variety of human cancer cell lines [5,44–47]. Moreover, simultaneous administration of the ROCK inhibitor Y-27632 and siCofilin1 lead to restore *FAT4* mRNA expression repressed by Y-27632 in the cells (Fig. 4B). Thus, these results suggest that Src-Cofilin1 axis plays an important role in the regulation of *FAT4* mRNA expression in breast cancer cells. Unexpectedly, the siRNA-mediated Cofilin knockdown did not completely block the reduction in *FAT4* mRNA expression by Src, although it abolished the inhibitory effect of Y-27632 on *FAT4* mRNA expression (Fig. 4B and 4E). Generally, Src regulates a wider array of signaling pathways than ROCK. As such, this may result in the significant differences presented in these data. Taken together, these results clearly show the inhibitory effect of Src on *FAT4* mRNA expression in response to actin dynamics during carcinogenesis.

The molecular mechanisms by which Src-induced actin depolymerization represses *FAT4* mRNA expression have not yet been elucidated. The *FAT4* gene is silenced by promoter hypermethylation in breast cancer cells [12]. Interestingly, Src elicits the overexpression of DNA methyltransferase (DNMT) 1 and induces DNA hypermethylation in rat 3Y1 fibroblasts [48]. Additionally, the MEK/Erk pathway induces DNA hypermethylation via the regulation of DNMT1 [49–51]. Therefore, Src-induced actin depolymerization has been speculated to repress *FAT4* mRNA expression via DNMT1-induced promoter hypermethylation during carcinogenesis, but further studies are needed for clarification.

Our study demonstrates that siRNA-mediated knockdown of FAT4 induces YAP/TAZ activity in MCF-10A cells (Fig. 6A and 6B). In line with this view, recent studies utilizing *in vivo* electroporation have demonstrated that Fat4 downregulates Yap activity leading to the inhibition of neural progenitor cell proliferation during murine and avian neurogenesis [25,26]. In addition, it has been demonstrated that deletion of Fat4 in mice causes primarily the nuclear accumulation of Yap in the nephron progenitor cells [52]. As such, these *in vivo* findings suggest that the Fat4-Hippo axis is highly conserved in mammals. However, the molecular underpinnings of Fat4-mediated nuclear translocation of Yap/Taz in mammals still remain unknown. Because there is no mammalian counterpart of *Drosophila* Dachs, which is regulated by Fat to activate the Hippo pathway [9,10,18], Fat4 should modulate Yap/Taz activity differently from Fat. In this regard, our research demonstrates that YAP phosphorylation levels (at least at Ser127) in FAT4 siRNA-treated cells are consistent with those found in control siRNA-treated MCF-10A cells (S7 Fig.). Nevertheless, MST1 kinase (mammalian Hippo ortholog) expression is reduced in FAT4 siRNA-treated cells (S7 Fig.). Accordingly, it is possible that nuclear localization of YAP is regulated by FAT4 through a Ser127 phosphorylation-independent mechanism.

It is also known that FAT4 is involved in the maintenance of planar cell polarity (PCP) [9]. Indeed, Fat4 mutant mice and PCP gene mutant mice show similar defects of the inner ear such as significant disruptions in orientation of hair cells and elongation of the cochlea [53]. Moreover, PCP genes have also been shown to regulate actin dynamics [54]. Collectively, these findings suggest that Fat4 regulates actin dynamics as well as vertebrate PCP. Interestingly, YAP/TAZ activity can be modulated by cytoskeletal tension induced by cell spreading in a Hippo pathway-independent manner [10,21,35]. Therefore, we can surmise that FAT4 regulates the YAP/TAZ activity through modulating actin cytoskeleton. In fact, we show that FAT4 knockdown induces abnormal spiny actin protrusions, which may enhance cell spreading (Fig. 6E). Thus, FAT4 may negatively regulate YAP/TAZ function by modulating actin cytoskeletal tension. Altogether, our current study presents a novel model in which FAT4 represses YAP/TAZ activity by regulating actin structures, at least in breast cancer cells. Further examination is needed to elucidate the molecular basis of YAP/TAZ inactivation by FAT4.

Accumulating evidences suggest the pathophysiological relevance between FAT4 reduction and carcinogenesis. Qi et al. have clearly demonstrated that *FAT4* mRNA is not expressed at all in many breast cancer cell lines (BT20, ZR75–1, and BT474 cells) [12]. We also found that MCF-7 breast cancer cells express far too low a level of *FAT4* mRNA compared with the MCF-10A normal mammary cell line (data not shown). Moreover, FAT4 knockdown has been shown to induce the malignant phenotype of human gastric cancer cell lines [55]. However, we show that FAT4 knockdown alone is not sufficient to induce cell proliferation or anchorage-independent growth in MCF-10A cells despite promoting YAP/TAZ activity (S6 Fig.; Fig. 6A and 6B); nevertheless, overexpression of YAP/TAZ can transform the cells [23,24]. Additionally, FAT4 knockdown alone does not induce cell proliferation in malignant breast cancer MDA-MB-231 cells (Fig. 3F). These data strongly demonstrate that cooperative effects between FAT4 reduction and other factors induce tumor transformation in MCF-10A cells. We propose that the MEK/Erk pathway involves in the Src-induced tumorigenesis as well as *FAT4* mRNA repression (Fig. 3A-D), because numerous studies have already established the importance of the MEK/Erk pathway for cell transformation [2,5,44,56,57]. Further comprehensive analyses are required to solve molecular mechanisms of Src-mediated carcinogenesis through repressing *FAT4* gene expression.

## Materials and Methods

### Cell culture and generation of stable cell lines

MCF-10A cells are immortalized normal human mammary epithelial cells that do not express the estrogen receptor (ER) [27,29]. MCF-10A cells were obtained from the American Type Culture Collection (ATCC). We generated MCF-10A cells stably expressing v-Src:ER chimeras (MCF-10A v-Src:ER) by using retroviruses and transformed them with 1 μM 4-Hydroxytamoxifen (H7904; Sigma) or the solvent ethanol as a control. MCF-10A cells were cultured in Assay Medium. Assay Medium is DMEM/F12 media (1:1) (11320–033; Gibco) supplemented with 2% horse serum (16050–122; Gibco), 0.5 μg/ml hydrocortisone (H4001; Sigma), 10 μg/ml insulin (093–06351; Wako), 100 ng/ml Cholera toxin (C8052; Sigma) and penicillin-streptomycin (15140–122; Gibco). In both siRNA knockdown experiments and Soft Agar Colony Formation Assays, Growth Medium was used as culture medium. Growth Medium is DMEM/F12 media (1:1) supplemented with 5% horse serum, 0.5 μg/ml hydrocortisone, 10 μg/ml insulin, 20 ng/ml epidermal growth factor (AF-100–15; PeproTech), 100 ng/ml Cholera toxin and penicillin-streptomycin. MDA-MB-231 cells, which are malignant mammary cancer cells, were cultured in DMEM/F12 media (1:1) supplemented with 10% FBS and penicillin-streptomycin. All cell lines were incubated at 37°C in a humidified atmosphere containing 5% CO_2_.

### Chemicals

The chemicals used in this study included U0126 (662005; Calbiochem), Y-27632 (257–00511; Wako), and Latrunculin A (sc-202691; Santa Cruz).

### Antibodies

The antibodies in this study included anti-α-tubulin (DM1A; Sigma), anti-Erk1/2 (137F5; Cell Signaling Technology), anti-Phospho-Erk1/2 (D13.14.4E; Cell Signaling Technology), anti-Cofilin (ACFL02; Cytoskeleton), anti-Phospho-Cofilin (Ser3) (#3311; Cell Signaling Technology), and anti-YAP (sc101199; Santa Cruz) (detects both YAP and TAZ).

### siRNA knockdown experiments

Cells were cultured in Growth Medium without antibiotics at 37°C for 24 h and were then transfected with siRNA using both Lipofectamine RNAiMAX (Invitrogen) and Opti-MEM I Reduced Serum Medium (31985–070; Gibco) according to the manufacturer’s protocol. The sequences of the siRNAs employed in this study are listed in S1 Table. The siRNA concentrations are described in the Figure Legends.

### RNA extraction and real-time quantitative PCR (RT-qPCR)

Total RNA was extracted from cells with ISOGENII (311–07361; Wako) or the RNeasy Mini Kit (Qiagen) and was subjected to cDNA synthesis with random hexamer primers and Moloney murine leukemia virus (M-MLV) reverse transcriptase (Invitrogen) according to the manufacturer’s protocols. RT-qPCR was performed with FastStart Universal SYBR (Roche) and a Thermal Cycler Dice Real Time System II (TP900; Takara Bio). The PCR primers that were used are listed in S2 Table. 18S rRNA was used for normalization.

### Western blotting

The cells were lysed in buffer containing 1% Triton X-100, 1% sodium deoxycholate, 0.1% SDS, 20 mM Tris-HCl, pH 7.5, 150 mM NaCl, 10 mM EDTA, 1 mM EGTA, 10% glycerol, 2.5 mM sodium pyrophosphate, 1 mM β-glycerophosphate, 1 mM sodium vanadate, and 1x protease inhibitor cocktail (Roche). Cellular debris was removed by centrifuging at 10 minutes at 13,000 xg at 4°C. Protein concentrations were determined with a Pierce BCA Protein Assay kit. Protein samples were loaded on 7%–12% SDS-polyacrylamide gels, separated, and transferred to Immobilon-P Membranes (IPVH00010; Merck). Membranes were blocked with Blocking One (03953–95; Nacalai tesque) and incubated with the antibodies indicated in the figures. The blots were treated with a horseradish peroxidase-conjugated secondary antibody (Invitrogen) and were developed with an enhanced chemiluminescence (ECL) kit (RPN2106; GE Healthcare). All original uncropped and unadjusted blots are shown in S8 Fig.

### Fluorescent staining

The cells were fixed with 4% formaldehyde solution in PBS on ice for 15 min, washed 3 times, and were permeabilized with 0.5% Triton X-100 in PBS for 10 min at room temperature (RT). After blocking cells with PBS containing 1% skim milk for 20 min at RT, the cells were incubated overnight at 4°C with anti-YAP antibody diluted in PBS containing 1% skim milk. After washing the cells 3 times with PBS, the cells were incubated with Alexa Fluor 546-conjugated secondary antibody (A-11030; Invitrogen) diluted in PBS containing 1% skim milk for 1 h at RT. To stain actin filaments (F-actin), the cells were blocked with PBS containing 5% BSA for 30 min at RT and were incubated with CF594 Phalloidin (00045; Biotium) in PBS containing 1% BSA for 20 min at RT, followed by PBS washes. The nuclei were stained with 4’,6’-diamidino-2-phenylindole (DAPI) (100 ng/ml) for 10 min at RT. Finally, the cells were sealed with fluorescence mounting medium (S3023; Dako). Fluorescent images were captured with an Olympus LX71 fluorescence microscope.

### WST-1 Assay

The cells were seeded at 4.0×10^3^ cells per well (96-well plate), cultured for 16 h and treated with TAM at 37°C for 48 h. For siRNA-mediated FAT4 knockdown experiments, MCF-10A cells were treated with FAT4 siRNA by reverse transfection according to the manufacturer’s protocol. The cells were seeded at 2.8×10^3^ cells per well (96-well plate) and incubated at 37°C for 48 h. On the other hand, MDA-MB-231 cells were seeded at 3.7×10^3^ cells per well (96-well plate), cultured for 24 h and treated with U0126 at 37°C for 48 h. For siRNA-mediated FAT4 knockdown experiments by reverse transfection, the cells were seeded at 2.5×10^3^ cells per well (96-well plate) and incubated at 37°C for 72 h or otherwise 24 h followed by treatment with U0126 for 48 h. Next, WST-1 reagent (5015944; Roche) was added to the cells for a 2 h incubation at 37°C. After shaking for 1 min, the viability of the cells was analyzed by a Microplate Reader (Molecular Devices) at 450 nm with a reference wavelength of 650 nm.

### Soft Agar Colony Formation Assay

The cells were seeded at 4.6×10^5^ cells (6 cm dish), cultured in Assay Medium at 37°C for 24 h and were then treated with TAM at 37°C for 72 h before the colony formation assay. For siRNA-mediated FAT4 knockdown experiments, the cells were seeded at 1.8×10^5^ cells (6 cm dish) and cultured in Growth Medium without antibiotics at 37°C for 24 h. The cells were then transfected twice with FAT4 or control siRNA (at 24 h and 48 h after plating) and cultured for a further 24 h after the last transfection. Subsequently, the cells were seeded at 7.0×10^2^ cells per well (96-well plate) in Growth Medium containing 0.4% agarose (100 μl) onto a lower layer of Growth Medium containing 0.5% agar (100 μl). Cells were fed 2 times per a week. The number of colonies was counted after 14 days.

### Cell Density Assay

The cells were seeded at 4.2×10^5^ cells (High density) or 7.0×10^4^ (Low density) cells per well (6-well plate), cultured in Assay Medium at 37°C for 24 h and subjected to RT-qPCR analysis.

### Softwell Assay

Twenty-four well Softwell plates of two different qualities of stiffness (50 kPa: stiff or 0.5 kPa: soft) (Matrigen) were coated by Collagen I, rat tail (354236, BD Biosciences) (10°g/ml) for 1 h at RT. The cells were seeded at 8.1×10^4^ cells per well in Softwell plates. The cells were cultured in Assay Medium for 48 h and subjected to RT-qPCR analysis.

### Statistical analyses

Statistical analyses were performed using Student’s t-test; a *P* < 0.05 was considered significant.

## Supporting Information

S1 Fig Transient activation of Src does not alter *TWIST1* gene expression. RT-qPCR analysis of *TWIST1* mRNA expression levels in TAM-treated MCF-10A v-Src:ER cells for the indicated times (mean ± SD, n = 3).
(TIF)

S2 Fig PD0325901, a MEK inhibitor, blocks the inhibitory effect of Src on *FAT4* mRNA expression in MCF-10A cells. RT-qPCR analyses of *FAT4* mRNA expression levels in TAM-treated cells following pretreatment with PD0325901 (444968; Calbiochem) (mean ± SD, n = 3).
(TIF)

S3 Fig Cofilin siRNA inhibits Latrunculin A-mediated reduction of *FAT4* mRNA expression. RT-qPCR analyses of *FAT4* mRNA expression levels in MCF-10A cells treated with 0.5 μM Latrunculin A (Lat. A) for 24 h following pretreatment with either control or Cofilin1 siRNA (siControl and siCofilin1, respectively, 20 nM) (mean ± SD, n = 3).
(TIF)

S4 Fig Phoshorylated-Cofilin expression is similar in high and low density MCF-10A cells. Western blotting for α-Tubulin and phosphorylated Cofilin in MCF-10A cells under high or low cell density conditions.
(TIF)

S5 Fig The effect of FAT4 knockdown on actin protrusions in low density MCF-10A cells. Staining for F-actin (Phalloidin) and nuclei (DAPI) in cells transfected with siControl or siFAT4 for 48 h. Abnormal spiny actin protrusions are indicated by white arrowheads. White bars, 50 μm.
(TIF)

S6 Fig The effects of FAT4 knockdown on cell proliferation and anchorage-independent growth. **A**. WST-1 Assay in MCF-10A cells after treatment with FAT4 siRNA for 48 h (siFAT4, 30 nM) (mean ± SD, n = 4). **B**. Soft Agar Colony Formation Assay in MCF-10A cells after treatment with siFAT4 for 72 h (30 nM) (mean ± SD, n = 6). Images show the cell colonies. Black bars, 1 mm.
(TIF)

S7 Fig FAT4 knockdown in MCF-10A cells does not alter phosphorylated YAP expression but reduces MST1 expression. Western blotting for phosphorylated YAP (Ser127) (#4911; Cell Signaling Technology), MST1 (#3682; Cell Signaling Technology), and α-Tubulin in MCF-10A cells. The cells were treated with control or FAT4 siRNA (siControl and siFAT4).
(TIF)

S8 Fig Original uncropped and unadjusted blots with molecular size markers. (TIF)

S1 Table Sequences of siRNAs. (TIF)

S2 Table Sequences of primers used for RT-qPCR. (TIF)
