# Supplementary figures and images for: Inhibitory Mechanism of FAT4 Gene Expression in Response to Actin Dynamics during Src-Induced Carcinogenesis
Source: PLoS One. 2015 Feb 13;10(2):e0118336. doi: 10.1371/journal.pone.0118336 (PMC4334522; doi:10.1371/journal.pone.0118336)

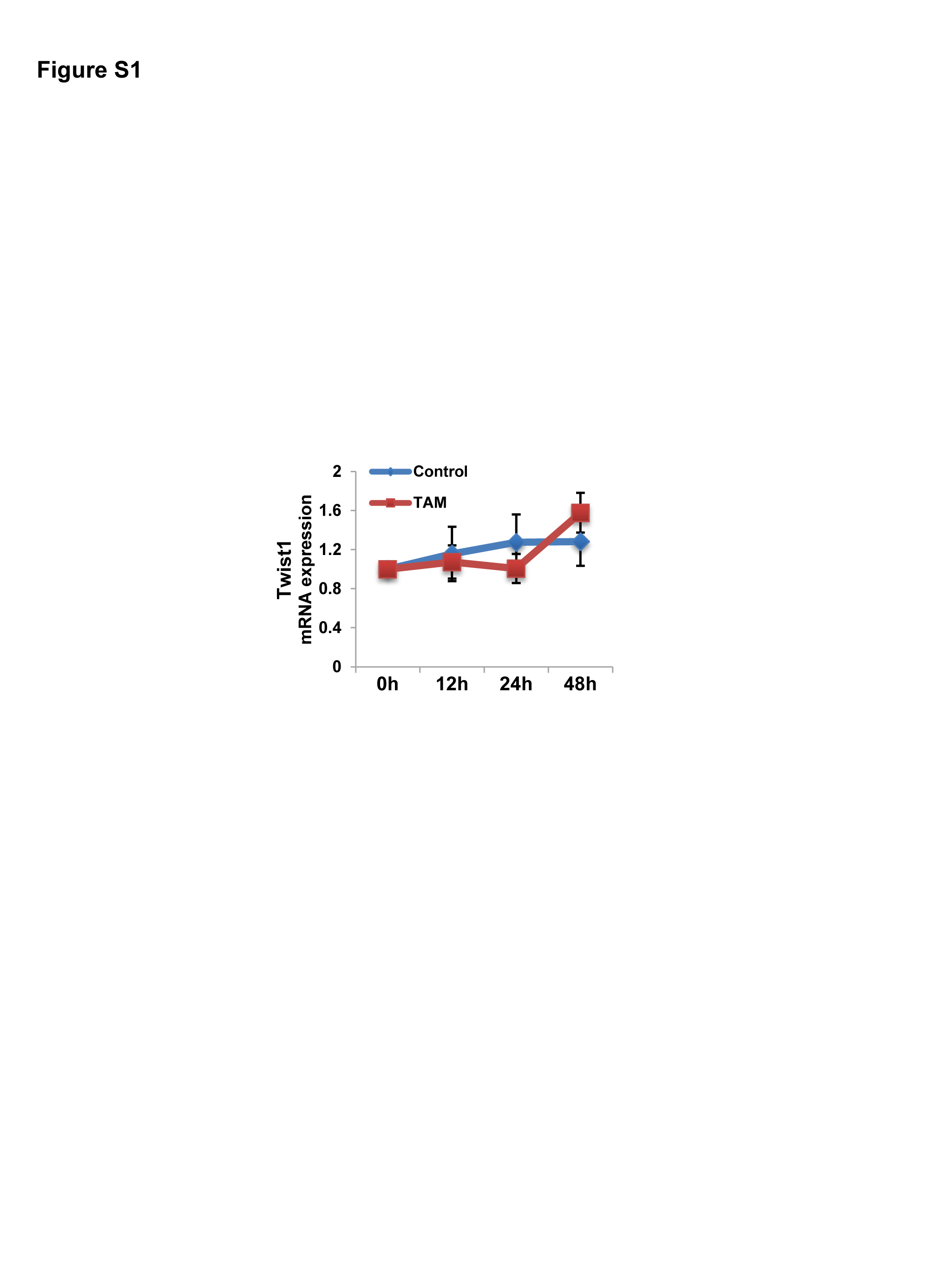

Supplement: S1 Fig — RT-qPCR analysis of TWIST1 mRNA expression levels in TAM-treated MCF-10A v-Src:ER cells for the indicated times (mean ± SD, n = 3). (TIF) [file pone.0118336.s001.tif]

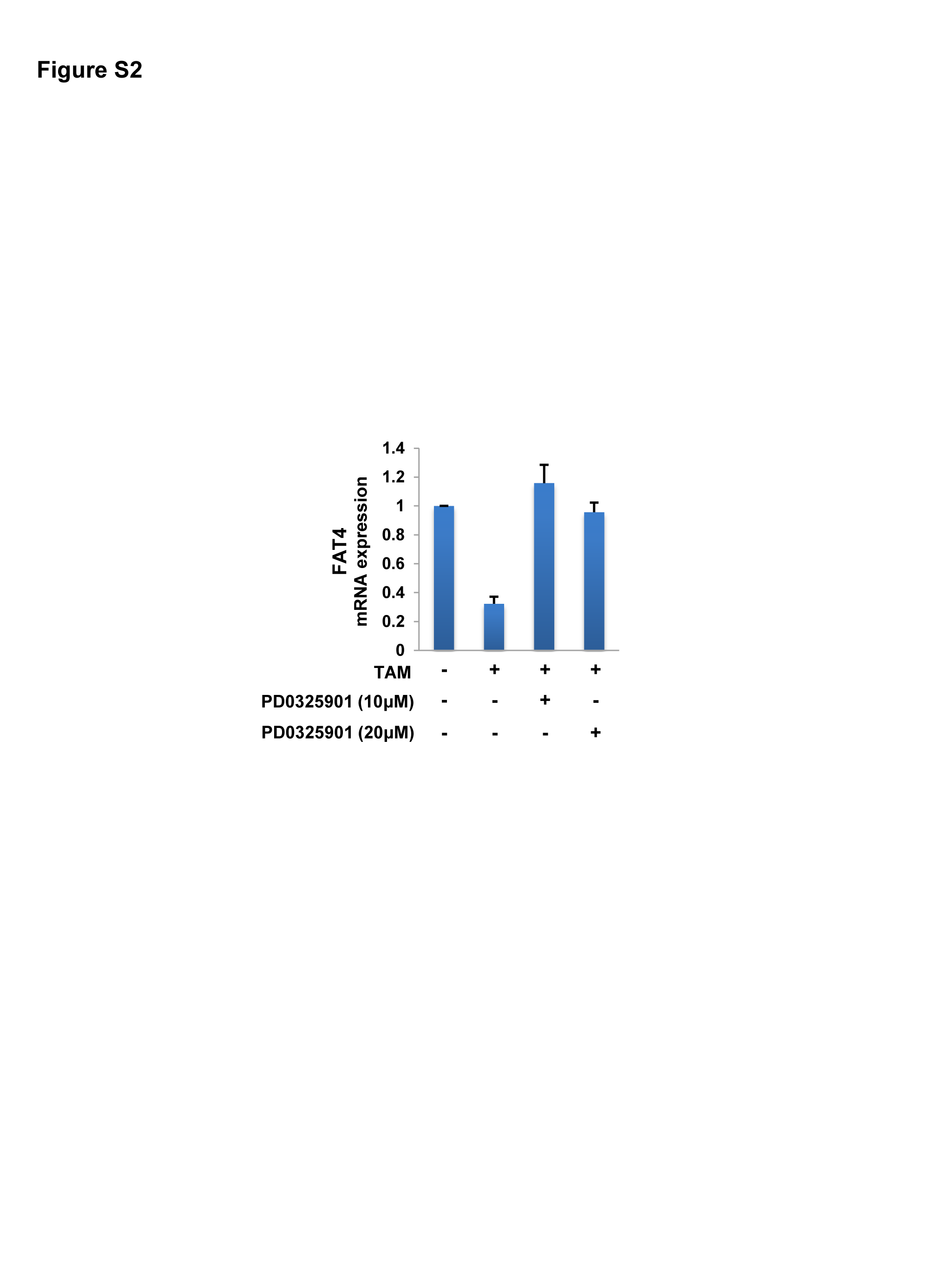

Supplement: S2 Fig — RT-qPCR analyses of FAT4 mRNA expression levels in TAM-treated cells following pretreatment with PD0325901 (444968; Calbiochem) (mean ± SD, n = 3). (TIF) [file pone.0118336.s002.tif]

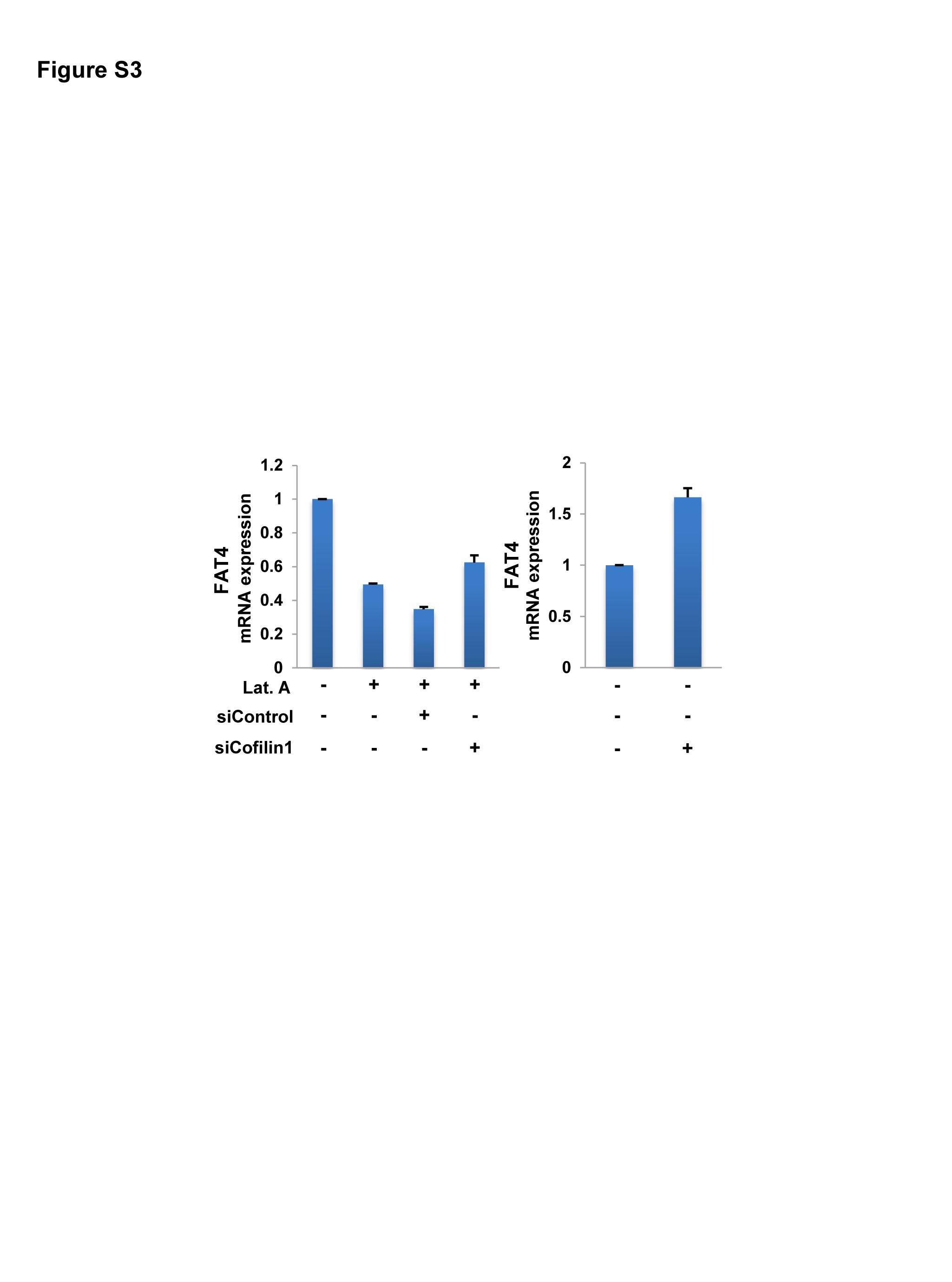

Supplement: S3 Fig — RT-qPCR analyses of FAT4 mRNA expression levels in MCF-10A cells treated with 0.5 μM Latrunculin A (Lat. A) for 24 h following pretreatment with either control or Cofilin1 siRNA (siControl and siCofilin1, respectively, 20 nM) (mean ± SD, n = 3). (TIF) [file pone.0118336.s003.tif]

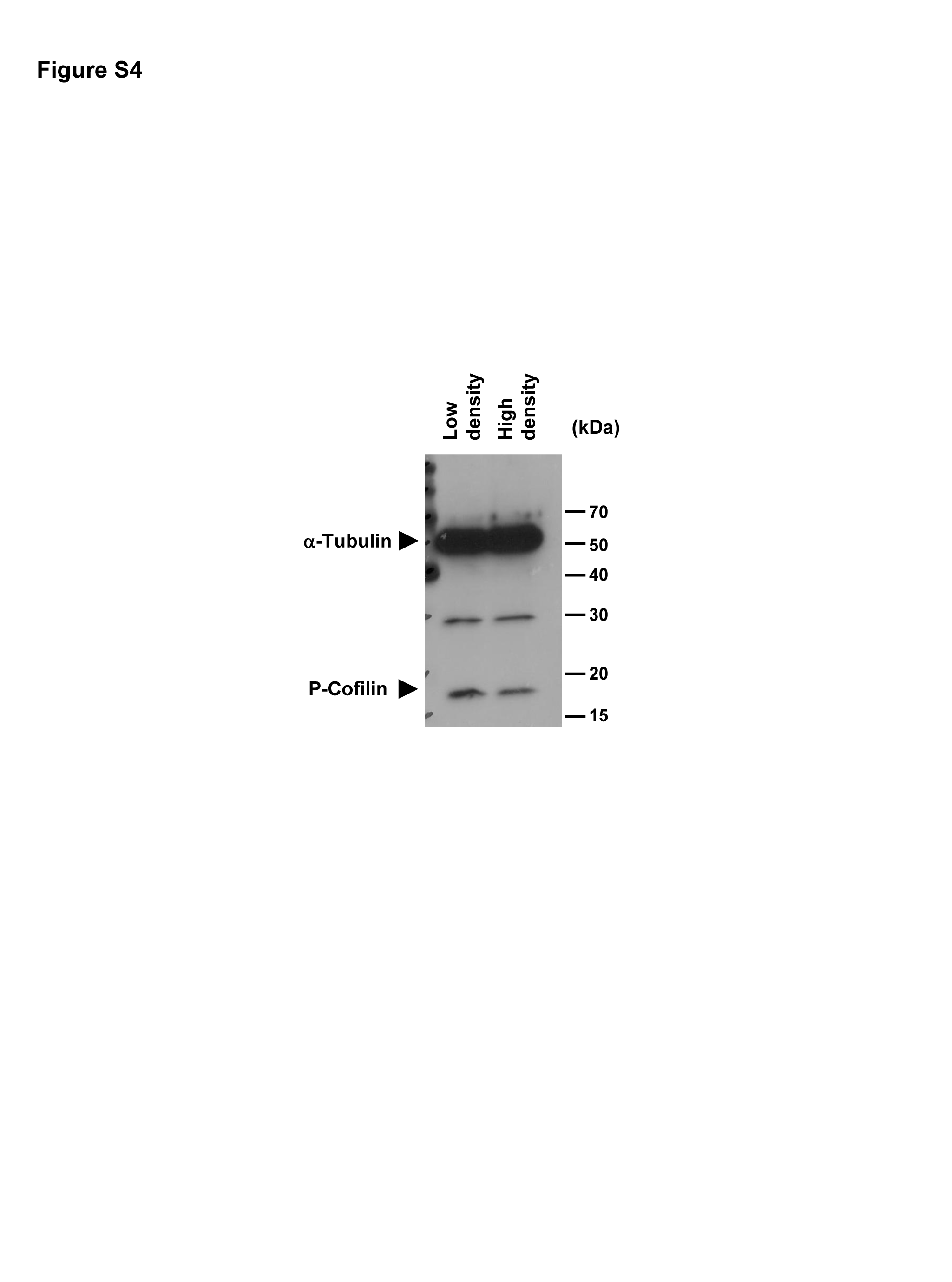

Supplement: S4 Fig — Western blotting for α-Tubulin and phosphorylated Cofilin in MCF-10A cells under high or low cell density conditions. (TIF) [file pone.0118336.s004.tif]

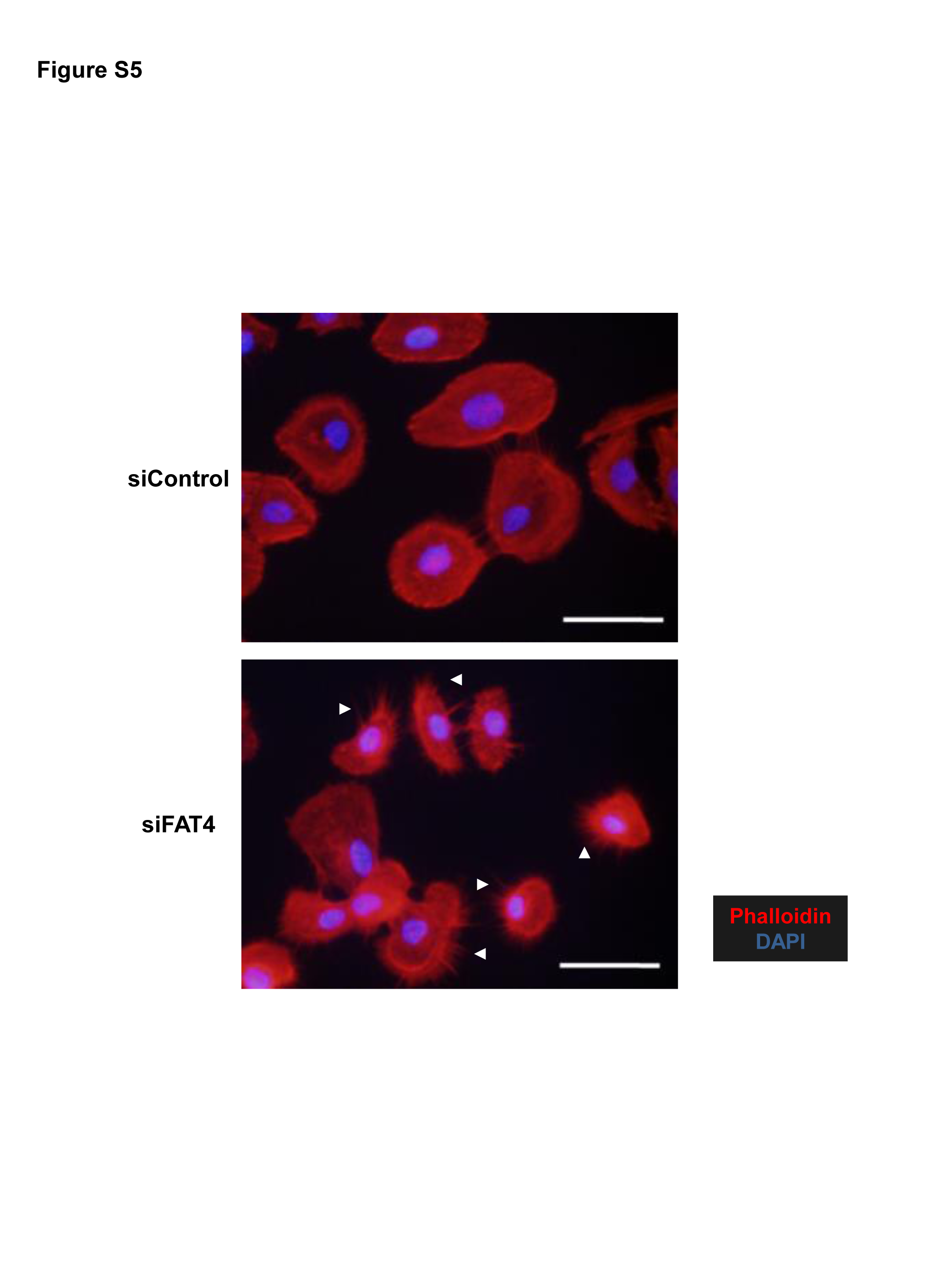

Supplement: S5 Fig — Staining for F-actin (Phalloidin) and nuclei (DAPI) in cells transfected with siControl or siFAT4 for 48 h. Abnormal spiny actin protrusions are indicated by white arrowheads. White bars, 50 μm. (TIF) [file pone.0118336.s005.tif]

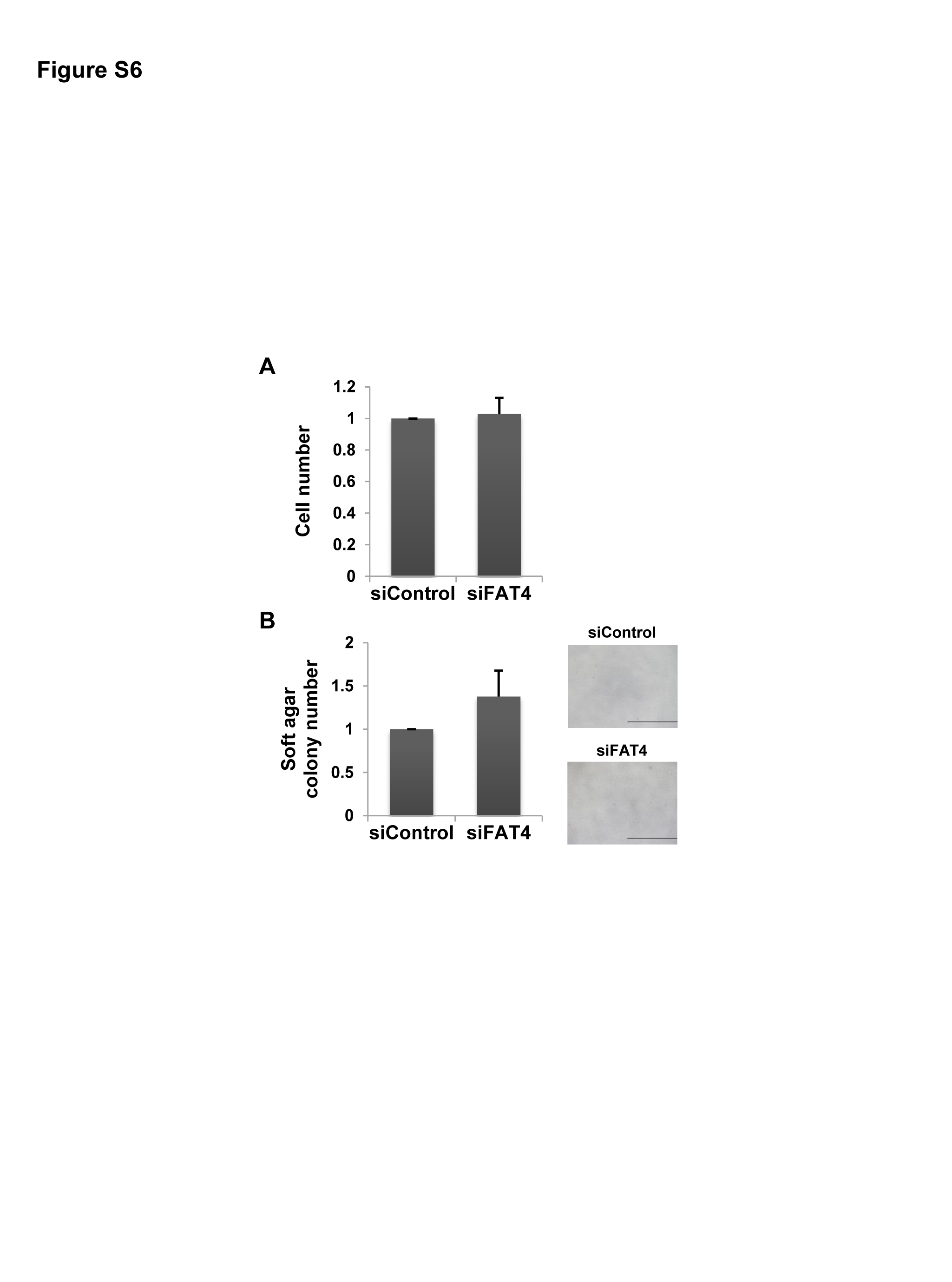

Supplement: S6 Fig — A. WST-1 Assay in MCF-10A cells after treatment with FAT4 siRNA for 48 h (siFAT4, 30 nM) (mean ± SD, n = 4). B. Soft Agar Colony Formation Assay in MCF-10A cells after treatment with siFAT4 for 72 h (30 nM) (mean ± SD, n = 6). Images show the cell colonies. Black bars, 1 mm. (TIF) [file pone.0118336.s006.tif]

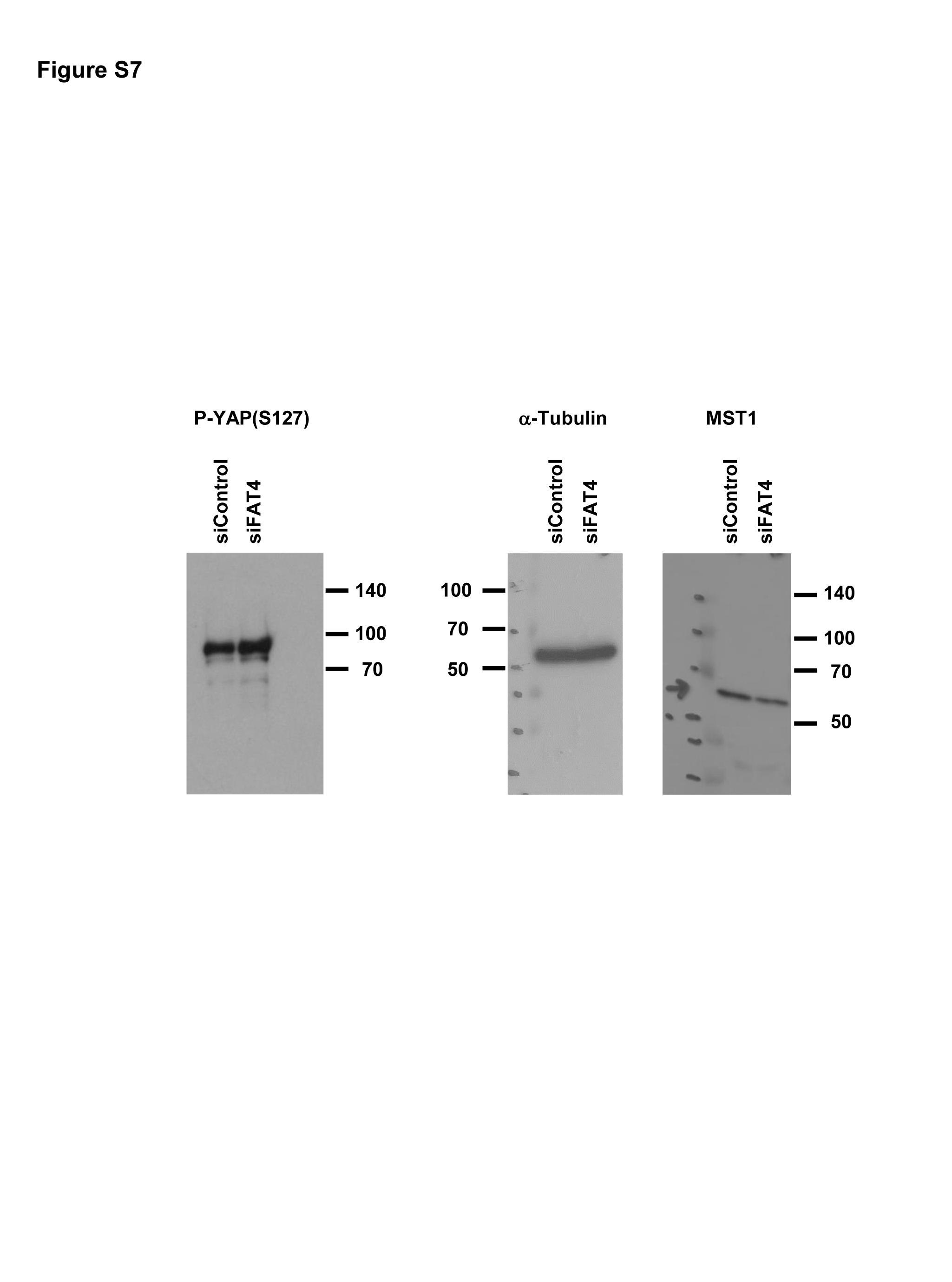

Supplement: S7 Fig — Western blotting for phosphorylated YAP (Ser127) (#4911; Cell Signaling Technology), MST1 (#3682; Cell Signaling Technology), and α-Tubulin in MCF-10A cells. The cells were treated with control or FAT4 siRNA (siControl and siFAT4). (TIF) [file pone.0118336.s007.tif]

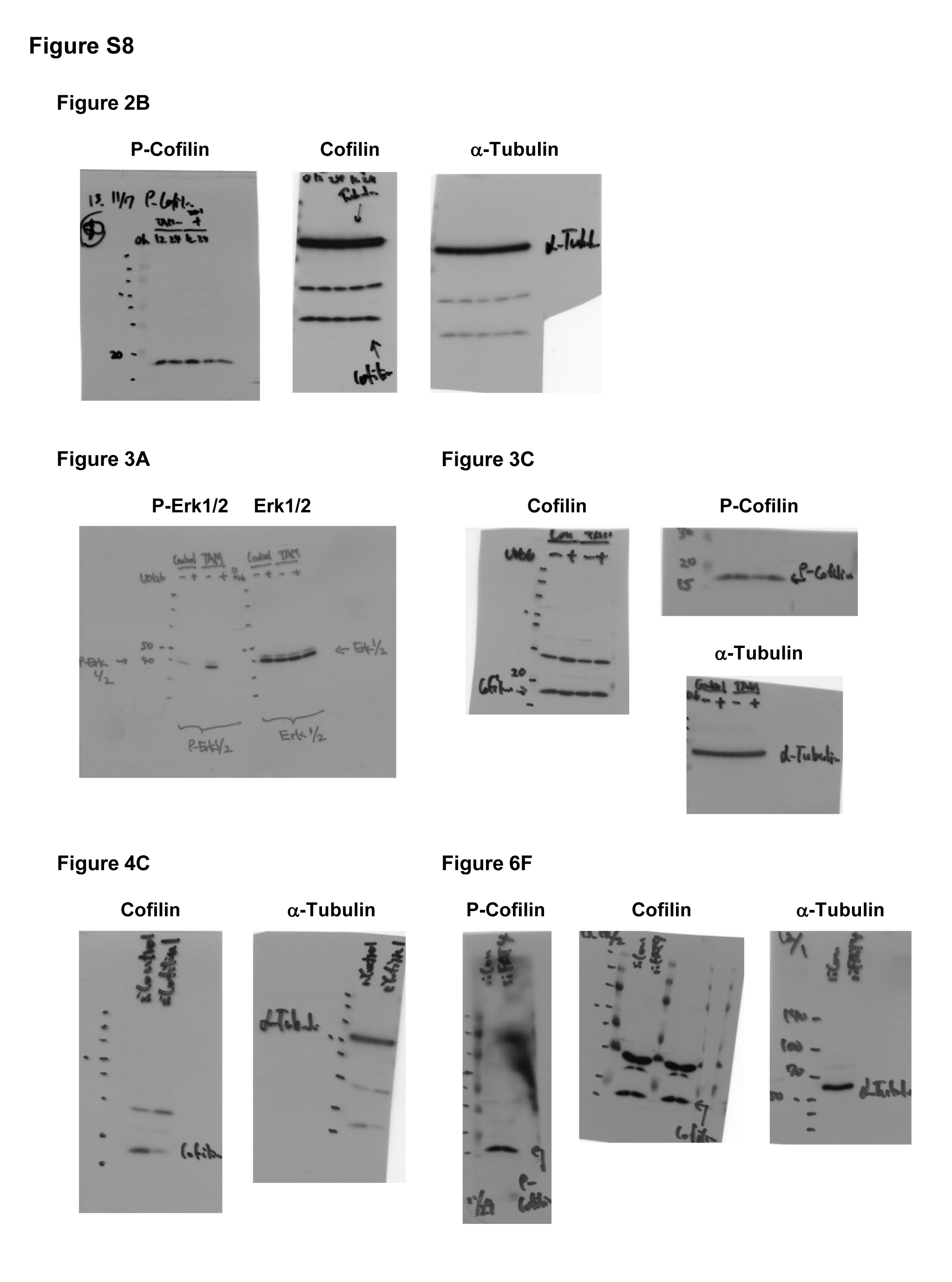

Supplement: S8 Fig — (TIF) [file pone.0118336.s008.tif]

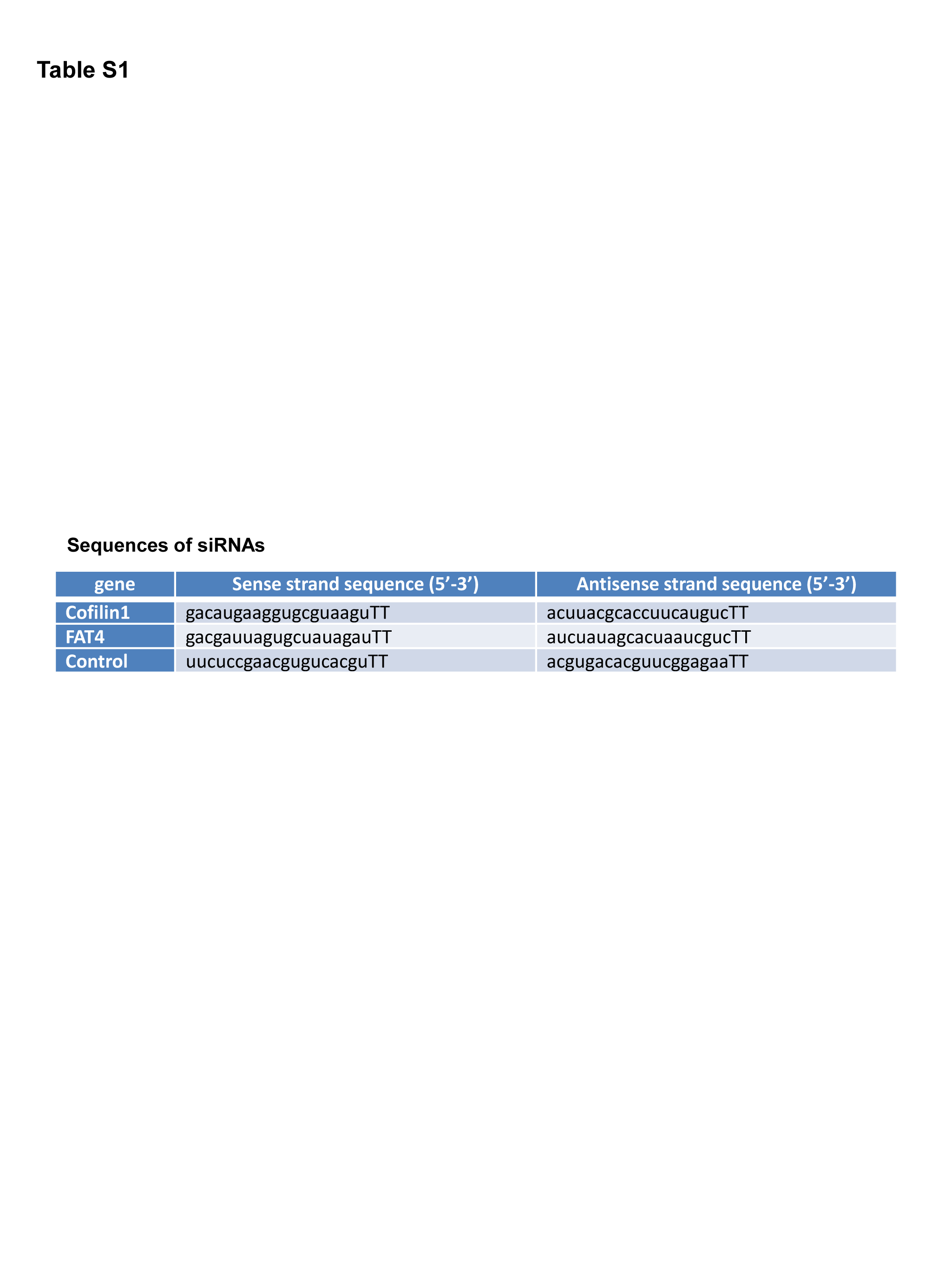

Supplement: S1 Table — (TIF) [file pone.0118336.s009.tif]

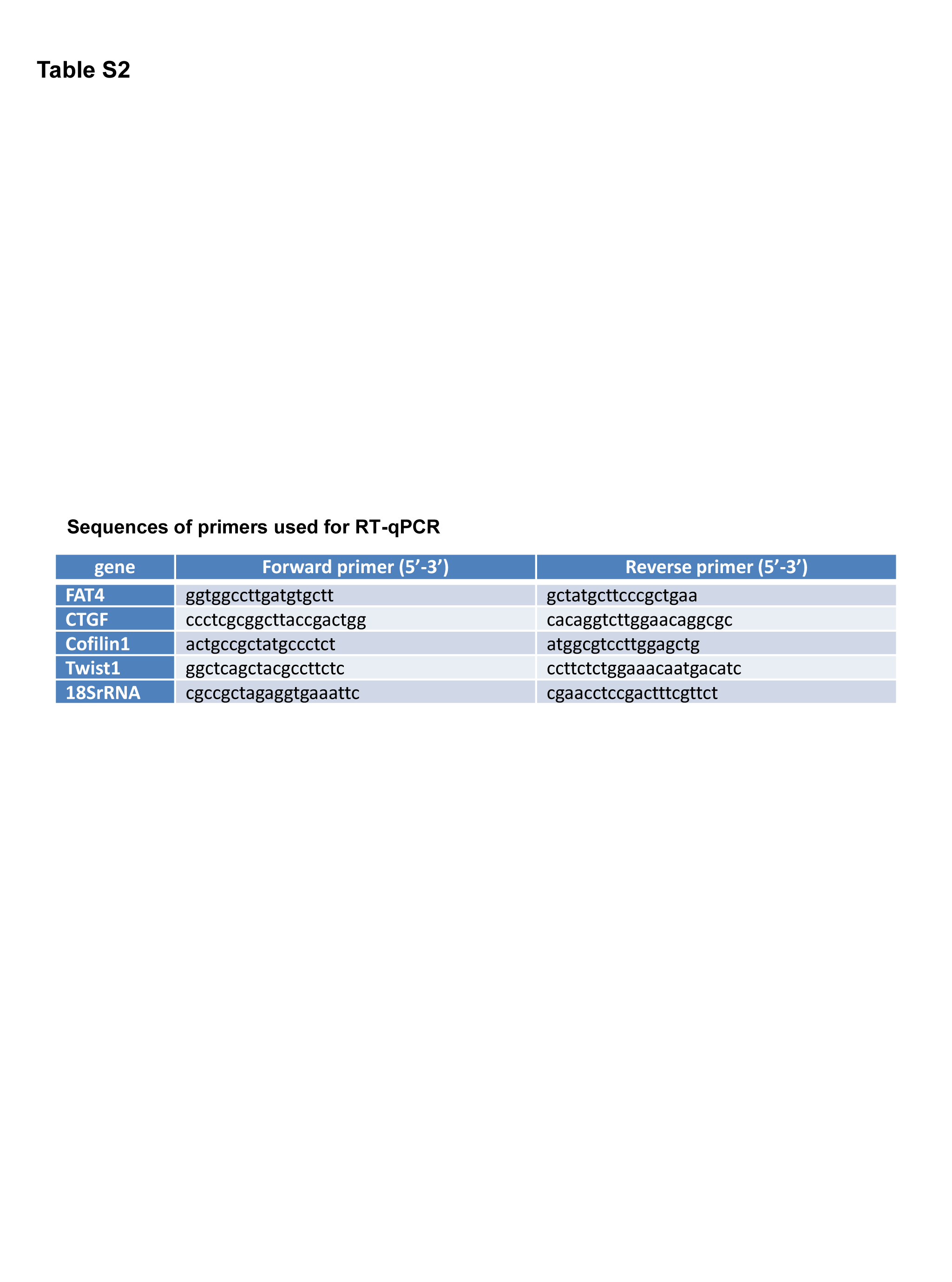

Supplement: S2 Table — (TIF) [file pone.0118336.s010.tif]
